# Complete mitochondrial genome of *Rhopalaea crassa* (Ascidiacea, Aplousobranchia, Diazonidae) and phylogenetic analysis

**DOI:** 10.1080/23802359.2026.2760061

**Published:** 2026-11-10

**Authors:** Soyeon Kwon, Taekjun Lee

**Affiliations:** a Marine Animal Biodiversity Center, Sahmyook University, Seoul, Korea; b Department of Environmental Horticulture, Graduate School of Sahmyook University, Seoul, Korea; c Department of Animal Resources Science, Sahmyook University, Seoul, Korea

**Keywords:** Tunicata, sea squirt, phylogeny, mitogenome, gene rearrangement

## Abstract

In this study, we determined the complete mitochondrial genome of *Rhopalaea crassa* (Herdman 1880), a solitary ascidian belonging to the family Diazonidae. The mitochondrial genome of *R. crassa* is 15,413 bp in length, containing 13 protein-coding genes, 24 transfer RNA genes, and two ribosomal RNA genes. The mitochondrial gene arrangement of *R. crassa* differs from those reported from previously sequenced Aplousobranchia mitogenomes. Phylogenetic analyses based on concatenated protein-coding genes consistently recovered *R. crassa* as the sister species of *R. idoneta* within Diazonidae, with strong support from both Maximum Likelihood and Bayesian Inference analyses.

## Introduction

*Rhopalaea* Philippi, 1843, includes both solitary and colonial ascidians in the family Diazonidae Seeliger, 1906, order Aplousobranchia Lahille, 1886, and comprises 21 extant species (WoRMS Editorial Board 2026). *Rhopalaea crassa* (Herdman 1880) is the only species of this genus recorded in South Korea (Seo 2021; MBRIS (Marine Bio-Resource Information System) 2026).

*Rhopalaea crassa* is a solitary ascidian characterized by a club-shaped body and conspicuous yellow patches in its transparent tunic (Nishikawa 1991; Kott 2009; Seo 2021) (Figure 1). Although *R. crassa* is distributed in tropical and subtropical waters of the Indo-West Pacific (Nishikawa 1991), no molecular data are available for this species. Within the genus, *Rhopalaea idoneta* is the only species for which a complete mitochondrial genome has been characterized (Shenkar et al. 2016), limiting comparative mitogenomic studies within Diazonidae.

**Figure 1.**
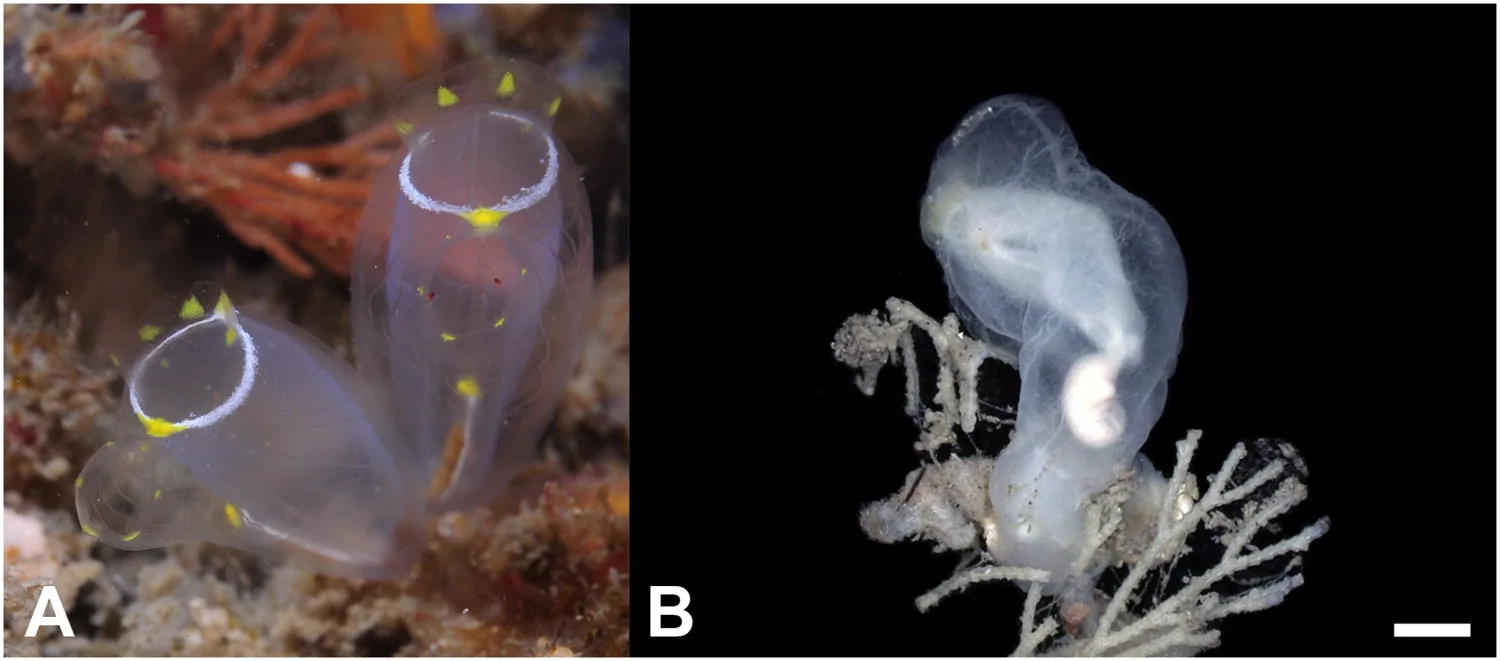
*Rhopalaea crassa* (Herdman 1880). (A) Live specimens attached to bedrock, photographed by Chanyong Park (Nikon D90; used with permission). (B) Specimen preserved in ethanol showing mantle and alimentary organs, photographed by Soyeon Kwon using a stereomicroscope (Leica S9i, Leica Microsystems). Scale bar = 2 mm.

We determined the first complete mitochondrial genome of *R. crassa* from Jejudo Island, South Korea, and analyzed its gene organization and phylogenetic relationships within Ascidiacea.

## Materials and methods

### Sample collection and species identification

Two individuals of *Rhopalaea crassa* were collected by trimix diving from Munseom Island, Jeju Island, South Korea (33°13′32.8”N, 126°34′08.6”E) at 55.6 m depth on 17 September 2025. They were transported alive to the laboratory.  Tissue was excised for DNA extraction, and the remaining specimens were preserved in 95% ethanol. They were deposited at Sahmyook University (https://sites.google.com/view/mbri/home; Taekjun Lee, leetj@syu.ac.kr) under the voucher number SYURC01.

Morphological observations were conducted using a stereomicroscope (Leica S9i: Leica Microsystems Co., Ltd., Wetzlar, Germany) with LAS V4.13 software. Taxonomic identification followed Nishikawa (1991) and Seo (2021).

### DNA extraction, generation of mitochondrial genome sequence, and annotation

Total genomic DNA was extracted from the whole-body tissue using a DNeasy^®^ Blood & Tissue Kit (Qiagen, Hilden, Germany). Partial mitochondrial cytochrome c oxidase subunit I (*COI)* sequences were amplified using dinF and Nux1R (Brunetti et al. 2017) under the PCR conditions in Table S1. Genomic DNA was further amplified using the REPLI-g Mitochondrial DNA Kit (Qiagen) with random primers (Thermo Fisher Scientific, Waltham, MA, USA).

Next-generation sequencing was conducted at the National Instrumentation Center for Environmental Management, Seoul National University, Seoul, Korea. A library prepared using the KAPA Hyper Prep Kit (Kapa Biosystems, Woburn, MA, USA) was paired-end sequenced on the Illumina NextSeq 1000 platform (Illumina Inc., San Diego, CA, USA). The mitochondrial genome was assembled de novo using NOVOPlasty v4.3.5 (Dierckxsens et al. 2017), with amplified *COI* as a seed. Raw sequencing reads were mapped to the assembled mitochondrial genome using Geneious Prime v.2026.0.2 (Biomatters, New Zealand). Sequencing depth was calculated from the resulting BAM file using SAMtools (Danecek et al. 2021) and visualized using RStudio (R Core Team 2026) (Figure S1).

The genome was annotated using MITOS2 (Al Arab et al. 2017; Donath et al. 2019) on the Galaxy server (Afgan et al. 2026). Transfer RNA (tRNA) gene annotations were further evaluated using ARWEN v1.2.3 (Laslett and Canbäck 2008) implemented in GeSeq (Tillich et al. 2017), and annotations were verified and refined in Geneious Prime v.2026.0.2 (Biomatters). The circular mitochondrial genome map was visualized using Proksee (Grant et al. 2023) (Figure 2).

**Figure 2.**
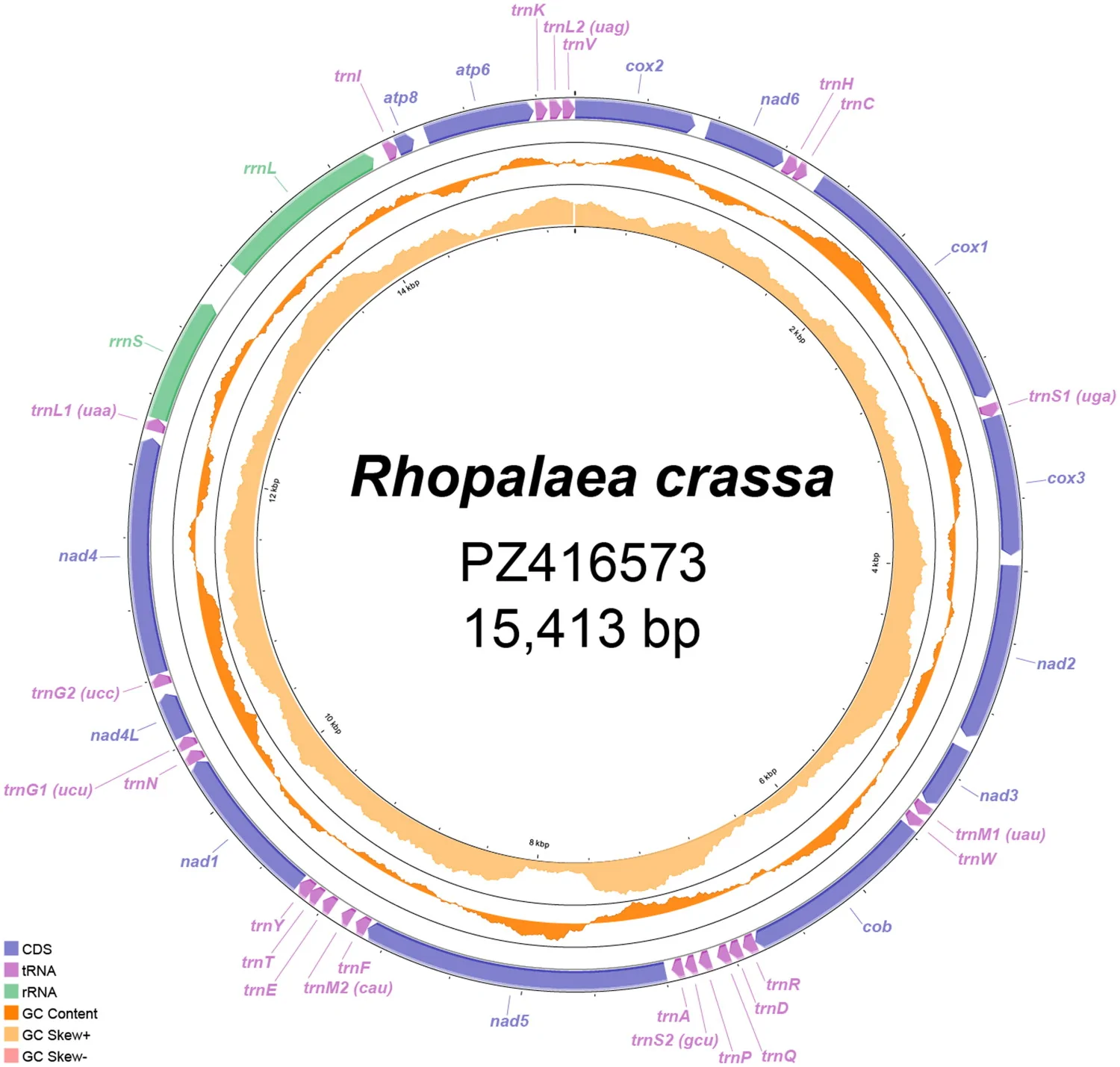
Circular complete mitochondrial genome map of *R. crassa* (PZ416573) in this study. The outer circle represents the arrangement of mitochondrial genes, including protein-coding genes (CDS), transfer RNA genes (tRNAs), and ribosomal RNA genes (rRNAs), shown in blue, purple, and green, respectively. The complete mitochondrial genome is 15,413 bp in length, with all genes encoded on the same strand. The inner plots indicate GC content and GC skew across the mitochondrial genome. Positive and negative GC skew values are shown in light orange and pink, respectively.

### Phylogenetic analysis

The dataset comprised 22 mitochondrial genome sequences representing *R. crassa* and 19 species retrieved from GenBank, including 10 sequences from Aplousobranchia, seven from Phlebobranchia, and three from Thaliacea, with two *Styela* Fleming, 1822 species (Stolidobranchia) as outgroups.

The sequences of 12 protein-coding genes (PCGs), excluding *atp8*, were aligned using MAFFT v7.490 (Katoh et al. 2002; Katoh and Standley 2013). The *atp8* gene was excluded because it was absent from some mitochondrial genomes included in the dataset (Stach et al. 2010; Smith et al. 2015). Both ribosomal RNA (rRNA) genes were also excluded because *rrnL* could not be reliably identified in the unannotated mitochondrial genome of *Diazona violacea* (OZ413588). The optimal substitution model for each gene was estimated using jModelTest v.2.1.10 (Darriba et al. 2012) (Table S2).

Maximum Likelihood (ML) analysis was performed using RAxML GUI 2.0 (Edler et al. 2021) with 1,000 bootstrap replicates. Bayesian Inference (BI) analysis was performed using MrBayes 3.2.7 (Ronquist and Huelsenbeck 2003) with two independent Markov chain Monte Carlo runs of four chains for 5,000,000 generations, sampled every 1,000 generations with the first 25% discarded as burn-in. Trees were visualized using FigTree v.1.4.4 (Rambaut 2009).

## Results

### *Complete mitochondrial genome structure of* R. crassa

The complete mitochondrial genome of *R. crassa* measured 15,413 bp in length (GenBank accession number PZ416573). It contained 13 PCGs, 24 tRNA genes, and two rRNA genes, all encoded on the H strand (Figure 2). Base composition was A (22.4%), T (47.5%), G (20.8%), and C (9.3%). The PCGs total 10,899 bp, and the 24 tRNA genes ranged from 63 bp (*trnC*) to 79 bp (*trnY*). Two genes each were found for *trnS*, *trnL*, *trnM*, and *trnG*; the additional *trnM* and *trnG*, relative to the typical metazoan complement, are consistent with previous reports in ascidians (Yokobori et al. 1999; Gissi et al. 2004).

Three start codons were detected (Table S2): ATG in six genes (*cox1*, *cox2*, *cox3*, *cob*, *nad4L*, and *atp8*), TTG in six (*nad1*, *nad2*, *nad3*, *nad4*, *nad5*, and *atp6*), and GTG in *nad6*. All PCGs concluded with TAG or TAA. The genome showed compact organization, with two gene overlaps (*trnT*–*trnY*, 4 bp; *trnL*–*rrnS*, 2 bp) and 34 intergenic non-coding regions (1–244 bp, total 1,155 bp), the longest between *rrnS* and *rrnL*.

### Phylogenetic analysis

ML and BI analyses of the 12 concatenated PCGs produced identical topologies (Figure 3). *Rhopalaea crassa* (PZ416573) was recovered as sister to *R. idoneta* (LN877970) within Diazonidae, with strong nodal support (bootstrap support (BS) = 100%, posterior probability (PP) = 1). The *Rhopalaea* clade formed a monophyletic group with *Diazona violacea* (OZ413588) within Diazonidae (BS = 100%, PP = 1), which was sister to *Aplidium conicum* (FN313538) (Polyclinidae) (BS = 100%, PP = 1). Clavelinidae (*Clavelina oblonga* (AM292604) and *C. lepadiformis* (AM292603, FJ839918)) formed a separate lineage, although the relationship among Aplousobranchia families showed lower support (BS = 55%, PP = 0.81). *Ciona* was recovered as sister to Aplousobranchia (BS = 83%, PP = 1), and Thaliacea was sister to this clade (BS = 72%, PP = 0.98). Overall, the phylogenetic results supported the placement of *R. crassa* within Aplousobranchia and supported a close relationship between the two available *Rhopalaea* mitochondrial genomes.

**Figure 3.**
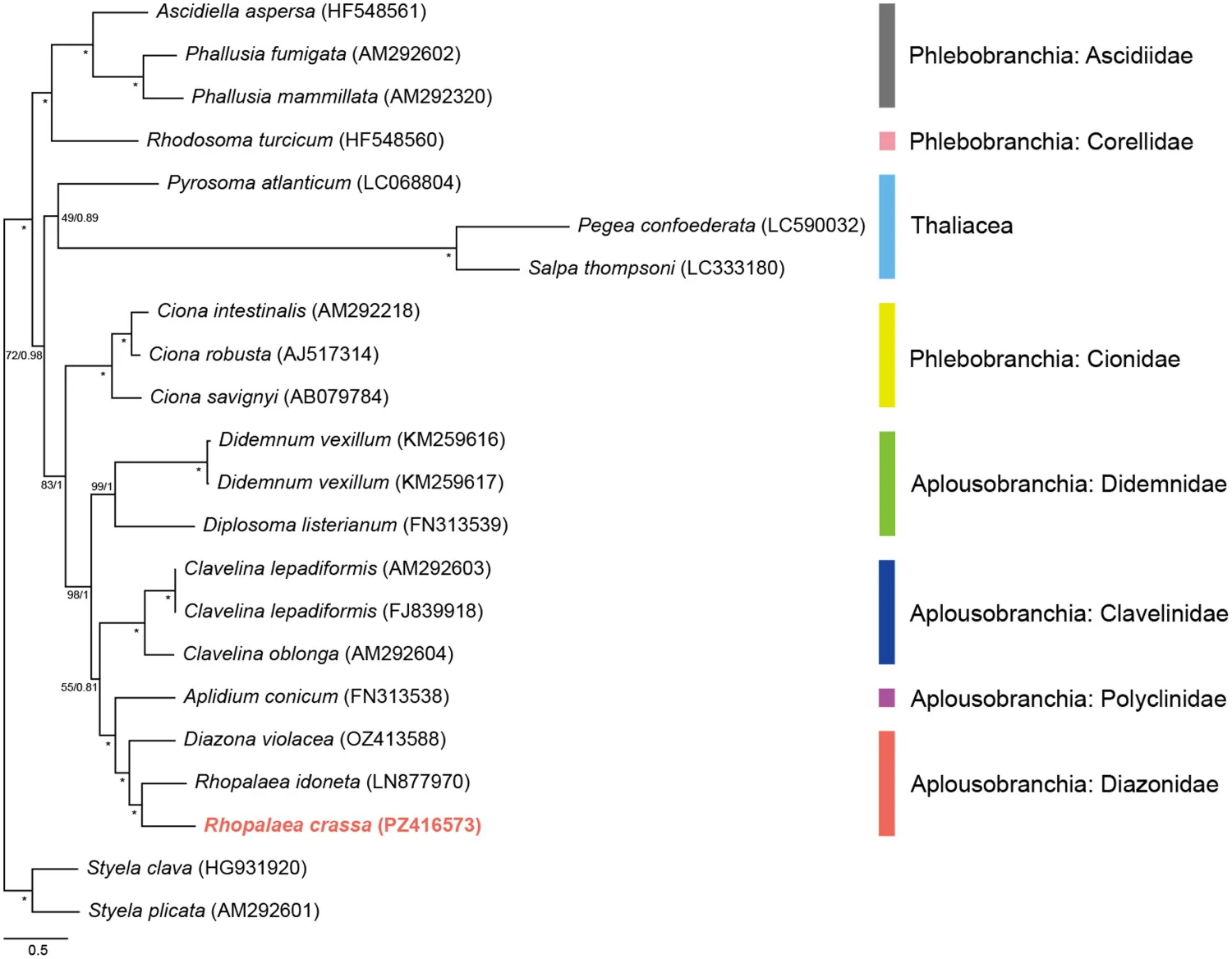
Phylogenetic tree based on the concatenated nucleotide sequences of 12 mitochondrial protein-coding genes (PCGs). The tree topology was reconstructed using Maximum Likelihood (ML) and Bayesian Inference (BI) analyses. Node support values are indicated as ML bootstrap support values and Bayesian posterior probabilities (BS/PP). An asterisk (*) indicates maximum support in both analyses (BS = 100%, PP = 1.0). The sequences analyzed were as follows: AB079784 (Yokobori et al. 2003); AJ517314 (Gissi et al. 2004); AM292218 (Iannelli et al. 2007b); AM292601, AM292603, FN313538, FN313539 (Gissi et al. 2010); AM292320, AM292602 (Iannelli et al. 2007a); AM292604, HG931920 (Griggio et al. 2014); HF548560, HF548561 (Rubinstein et al. 2013); FJ839918 (Stach et al. 2010); KM259616, KM259617 (Smith et al. 2015); LC068804, LC590032, OZ413588 (Unpublished); LC333180 (Goodall-Copestake 2018); LN877970 (Shenkar et al. 2016).

## Discussion and conclusion

*Rhopalaea crassa* was recovered as sister to *R. idoneta*, and *Rhopalaea* clade was sister to colonial *Diazona violacea*, supporting the monophyly of the sampled Diazonidae (BS = 100%, PP = 1). As in Shenkar et al. (2016), Diazonidae was nested within Aplousobranchia as sister to *Aplidium* Savigny, 1816. The inclusion of *D. violacea* also addresses their suggestion that colonial Diazonidae be analyzed to confirm the monophyly of the family. *Ciona* was recovered as sister to Aplousobranchia (BS = 83%, PP = 1), rendering Phlebobranchia paraphyletic, consistent with Shenkar et al. (2016). Delsuc et al. (2018) recovered a similar topology with high posterior probability but low gene-jackknife support, whereas Kocot et al. (2018) did not recover *Ciona* as sister to Aplousobranchia and found Phlebobranchia to be either monophyletic or paraphyletic depending on the dataset. Thus, relationships between Phlebobranchia and Aplousobranchia remain unresolved. Thaliacea was recovered among the traditional ascidian lineages, consistent with previous phylogenomic studies (Delsuc et al. 2018; Kocot et al. 2018). However, the clade comprising Thaliacea, *Ciona*, and Aplousobranchia received moderate bootstrap support (BS = 72%), despite a high posterior probability (PP = 0.98).

The gene arrangements of *R. crassa* and *R. idoneta* differed considerably, sharing only four adjacent gene pairs (*trnS*–*cox3*, *trnW*–*cob*, *trnY*–*nad1*, and *trnL*–*rrnS*), consistent with the high variability of mitochondrial gene order in ascidians (Singh et al. 2009; Gissi et al. 2010; Stach et al. 2010). In *R. crassa*, GTG was used as the initiation codon of *nad6*, reflecting variability in start codon usage among ascidians (Yokobori et al. 1999), whereas all PCGs terminated with the standard stop codons TAA or TAG.

This study provides the first complete mitochondrial genome of *R. crassa*, expanding the mitogenomic resources for *Rhopalaea* and contributing to understanding mitochondrial genome evolution and phylogenetic relationships within Aplousobranchia.

## Supplementary Material

supplementary table2_revised.docx

supplementary table1_revised.docx

## Data Availability

The genome sequence data that support the findings of this study are openly available in GenBank of NCBI at [https://www.ncbi.nlm.nih.gov] under the accession no. PZ416573. The associated BioProject, SRA, and BioSample numbers are PRJNA1490973, SRR39500215, and SAMN61444107, respectively.
