# Supplementary material for: Complete mitochondrial genome of Rhopalaea crassa (Ascidiacea, Aplousobranchia, Diazonidae) and phylogenetic analysis
Source: Mitochondrial DNA B Resour. 2026 Nov 10;11(12):1400–4. doi: 10.1080/23802359.2026.2760061 (PMC13757907; doi:10.1080/23802359.2026.2760061)
Supplement: Supplementary file 1 [file TMDN_A_2760061_SM4034.docx]

**Table legends**

Table S1. PCR cycling conditions used for amplification of the mitochondrial *COI* sequences.

| **Stage** | **Temperature (°C)** | **Time** | **Number of cycles** |
| --- | --- | --- | --- |
| Initial denaturation | 95 | 5 min | 1 cycle |
| Denaturation | 95 | 30 sec | 35 cycles |
| Annealing | 46 | 50 sec |  |
| Extension | 72 | 50 sec |  |
| Final extension | 72 | 5 min | 1 cycle |
