# Supplementary material for: Complete mitochondrial genome of Rhopalaea crassa (Ascidiacea, Aplousobranchia, Diazonidae) and phylogenetic analysis
Source: Mitochondrial DNA B Resour. 2026 Nov 10;11(12):1400–4. doi: 10.1080/23802359.2026.2760061 (PMC13757907; doi:10.1080/23802359.2026.2760061)
Supplement: Supplementary file 2 [file TMDN_A_2760061_SM4035.docx]

**Table legends**

Table S2. Summary of substitution models used in the phylogenetic analysis and protein-coding gene characteristics of *R. crassa*. The phylogenetic dataset comprised 22 tunicate mitochondrial genome sequences (19 Ascidiacea and three Thaliacea). 12 protein-coding genes (PCGs), excluding *atp8*, were included in the phylogenetic analysis. The best-fit substitution models for each gene were selected using jModelTest under both AIC and BIC. Gene lengths (bp), start codons, and stop codons were determined from the *R. crassa* mitochondrial genome newly sequenced in this study.

| **No.** | **Gene** | **AIC** | **BIC** | **bp** | **Start Codons** | **Stop Codons** |
| --- | --- | --- | --- | --- | --- | --- |
| 1 | *cox2* | GTR+I+G | GTR+I+G | 684 | ATG | TAG |
| 2 | *nad6* | TVM+I+G | GTR+G | 462 | GTG | TAA |
| 3 | *cox1* | TVM+I+G | GTR+I+G | 1,554 | ATG | TAA |
| 4 | *cox3* | GTR+G | GTR+G | 789 | ATG | TAG |
| 5 | *nad2* | GTR+I+G | GTR+I+G | 1,002 | TTG | TAA |
| 6 | *nad3* | TVM+I+G | HKY+I+G | 369 | TTG | TAA |
| 7 | *cob* | GTR+I+G | GTR+I+G | 1,089 | ATG | TAG |
| 8 | *nad5* | TVM+I+G | GTR+I+G | 1,728 | TTG | TAG |
| 9 | *nad1* | GTR+I+G | GTR+I+G | 900 | TTG | TAG |
| 10 | *nad4L* | TVM+I+G | GTR+G | 258 | ATG | TAA |
| 11 | *nad4* | TVM+I+G | GTR+I+G | 1,341 | TTG | TAG |
| 12 | *atp*8 | - | - | 102 | ATG | TAA |
| 13 | *atp6* | GTR+I+G | GTR+I+G | 621 | TTG | TAG |
